# Zika-exposed microcephalic neonates exhibit higher degree of inflammatory imbalance in cerebrospinal fluid

**DOI:** 10.1038/s41598-021-87895-4

**Published:** 2021-04-19

**Authors:** Gustavo C. Nascimento-Carvalho, Eduardo C. Nascimento-Carvalho, Clara L. Ramos, Ana-Luisa Vilas-Boas, Otávio A. Moreno-Carvalho, Caian L. Vinhaes, Beatriz Barreto-Duarte, Artur T. L. Queiroz, Bruno B. Andrade, Cristiana M. Nascimento-Carvalho

**Affiliations:** 1 Bahiana Foundation for Science Development, Bahiana School of Medicine, Salvador, Bahia 40290-000 Brazil; 2 Cerebrospinal Fluid Laboratory, José Silveira Foundation, Salvador, Bahia 40170-100 Brazil; 3 Gonçalo Moniz Institute, Oswaldo Cruz Foundation, Salvador, Bahia 40296-710 Brazil; 4 Multinational Organization Network Sponsoring Translational and Epidemiological Research (MONSTER) Initiative, Salvador, Bahia 40296-710 Brazil; 5 University Salvador (UNIFACS), Laureate Universities, Salvador, Bahia 41820-021 Brazil; 6 School of Medicine, Faculdade de Tecnologia e Ciências (Uni-FTC), Salvador, Bahia 41741-590 Brazil; 7 Wellcome Centre for Infectious Disease Research in Africa, Institute of Infectious Disease and Molecular Medicine, University of Cape Town, Cape Town, 7700 South Africa; 8 Division of Infectious Diseases, Department of Medicine, Vanderbilt University School of Medicine, Nashville, TN 37232 USA; 9 Department of Pediatrics, Federal University of Bahia School of Medicine, Salvador, Bahia 40210-630 Brazil

**Keywords:** Immunology, Biomarkers

## Abstract

Not every neonate with congenital Zika virus (ZIKV) infection (CZI) is born with microcephaly. We compared inflammation mediators in CSF (cerebrospinal fluid obtained from lumbar puncture) between ZIKV-exposed neonates with/without microcephaly (cases) and controls. In Brazil, in the same laboratory, we identified 14 ZIKV-exposed neonates during the ZIKV epidemic (2015–2016), 7(50%) with and 7(50%) without microcephaly, without any other congenital infection, and 14 neonates (2017–2018) eligible to be controls and to match cases. 29 inflammation mediators were measured using Luminex immunoassay and multidimensional analyses were employed. Neonates with ZIKV-associated microcephaly presented substantially higher degree of inflammatory perturbation, associated with uncoupled inflammatory response and decreased correlations between concentrations of inflammatory biomarkers. The groups of microcephalic and non-microcephalic ZIKV-exposed neonates were distinguished from the control group (area under curve [AUC] = 1; P < 0.0001). Between controls and those non-microcephalic exposed to ZIKV, IL-1β, IL-3, IL-4, IL-7 and EOTAXIN were the top CSF markers. By comparing the microcephalic cases with controls, the top discriminant scores were for IL-1β, IL-3, EOTAXIN and IL-12p70. The degree of inflammatory imbalance may be associated with microcephaly in CZI and it may aid additional investigations in experimental pre-clinical models testing immune modulators in preventing extensive damage of the Central Nervous System.

## Introduction

A widespread epidemic of Zika virus (ZIKV) infection was reported in South America in 2015^1^. In 2016, a causal relationship between ZIKV infection during pregnancy and microcephaly was strongly suspected^2^, when a serious epidemic sparked a major concern given the hundreds of microcephalic neonates born in Brazil^3^. Subsequently, several studies have shown that ZIKV has high Central Nervous System (CNS) tropism and is harmful when the CNS is immature^4^.

In 2017, microcephaly was revealed on postmortem examination of 7 neonates with congenital ZIKV infection (CZI)^5^. Leptomeningeal and cerebral parenchymal inflammation was described with varying intensity and distribution^5^. In 2018, an immunohistochemical analysis of neural parenchyma tissues from 8 deceased neonate/stillbirth babies, 4 with and 4 without ZIKV infection, showed significantly higher expression of several cytokines receptors in ZIKV-positive microcephaly cases, suggesting that inflammation activation can aggravate the neuroinflammatory response and consequently increase CNS damage in neonates with fetal neural ZIKV infection and microcephaly^6^. Although extensive research has been done focusing on understanding the mechanisms of ZIKV-induced congenital microcephaly, there are still gaps. In a case-series of neonates with laboratory-confirmed CZI, 13% were born without microcephaly^7^. Additionally, neonates exposed to ZIKV during fetal life may be born with increased protein concentrations in cerebrospinal fluid (CSF), particularly in cases with microcephaly or abnormal neuroimaging findings^8^. A previous investigation enrolling 6 ZIKV-infected pregnant women who delivered during the study course showed that infants with congenital CNS deformities had significantly higher levels of IL-18 and IP-10 but lower levels of hepatocyte growth factor than those without such abnormalities born to ZIKV-infected mothers^9^. In a recent study that investigated concentrations of mediators of inflammation in umbilical cord blood of neonates, Vinhaes et al. have demonstrated that individuals with ZIKV-associated microcephaly exhibit an increased systemic inflammation profile compared to those that were exposed to ZIKV but did not develop such clinical outcome^10^. Whether imbalanced immune activation in peripheral blood is associated with changes in inflammation in CSF in the context of ZIKV microcephaly is still unknown.

In this context, we compared concentrations of inflammatory mediators in the CSF of neonates exposed to ZIKV during pregnancy (cases), with or without microcephaly, and neonates who were not exposed to ZIKV during pregnancy, without other congenital infection and without microcephaly (control group).

## Results

### Study population

Out of 16 cases, 2 did not have enough CSF sample. Then, 14 remained in our study, 7 (50%) with and 7 (50%) without microcephaly, 11 likely ZIKV-infected during the 1st trimester and 3 likely ZIKV-infected during the 2nd trimester of pregnancy. Out of these 3 cases, 2 did and 1 did not have microcephaly. Overall, 85 neonates were evaluated to be included in the control group, out of which 61 (71.8%) were excluded due to: age > 4 days (n = 45), CSF WBC count > 8/mm^3^ (n = 19), CSF protein > 132 mg/dL (n = 9), CSF RBC count > 1000/mm^3^ (n = 8), and ventricular tap (n = 1). Among 24 neonates further evaluated after interviewing mothers, 10 (41.7%) were excluded due to untreated maternal syphilis (n = 9) and CNS hemorrhage (n = 1). The reasons for performing LP in controls were: sepsis (n = 6), maternal syphilis (n = 5), seizure (n = 1), fever without source (n = 1), and acute maternal cytomegalovirus infection (n = 1). Congenital syphilis and congenital cytomegalovirus infections were ruled out as all of them had negative syphilis serological tests (all mothers had been treated for syphilis during pregnancy) and the neonate born to the woman with gestational acute cytomegalovirus infection had urine negative cytomegalovirus polymerase chain reaction during the first week of life. Therefore, the study group comprised 14 cases and 14 controls. Their baseline characteristics are presented in Supplementary Table S1.

### Inflammatory profile

Table 1 shows the distribution of inflammation mediators per distinct groups. When cases with and without microcephaly were compared IL-4 and TNF-β were significantly higher and MCP1 (CCL2) was significantly lower among microcephalic cases. When cases (with or without microcephaly) were compared with controls, IL-1α, IL-7, IP10 (CXCL10), and GCSF were significantly higher among controls. When only cases with microcephaly were compared with controls, IL-4 was significantly higher among cases. When only cases without microcephaly were compared with controls, IL-1α and IP10 (CXCL10) were significantly higher among controls.

**Table 1:** Comparison of CSF inflammatory parameters (median [p25th-p75th]) between neonates exposed to Zika virus during fetal life, with or without congenital microcephaly, and controls.

CSF inflammatory parameters (pg/ml)	Controls n = 14	Cases with and without microcephaly n = 14	P^a^	Cases with microcephaly n = 7	P^b^	Cases without microcephaly n = 7	P^c^	P^d^
**Results with significant difference**								
IL-1α	31.0 (20.6–39.3)	18.2 (13.8–22.5)	0.008	20.5 (15.5–33.0)	0.2	15.0 (13.0–20.0)	0.004	0.2
IL-7	13.3 (11.9–14.4)	11.5 (9.8–13.5)	0.048	11.5 (10.0–12.5)	0.07	11.4 (9.0–13.9)	0.2	0.9
IP10/CXCL10	1425.5 (657.8–2274.6)	447.8 (182.5–1358.0)	0.03	564.0 (260.0–2576.0)	0.5	261.5 (151.0–572.0)	0.006	0.3
MCP1/CCL2	10,611.8 (7253.5–14,501.9)	10,299.0 (6877.0–15,641.1)	0.7	7999.5 (3737.5–11,432.0)	0.2	15,580.0 (9166.0–16,028.0)	0.06	0.03
TNF-β	8.0 (7.4–9.6)	8.7 (7.1–9.7)	0.9	9.7 (8.5–10.0)	0.2	7.6 (6.5–8.8)	0.2	0.03
GCSF	16.0 (12.9–19.6)	13.0 (11.0–14.3)	0.047	13.0 (13.0–14.0)	0.2	11.0 (9.0–15.0)	0.06	0.2
IL-4	10.0 (9.5–10.7)	10.5 (9.4–11.6)	0.4	11.5 (10.2–13.0)	0.01	9.4 (8.4–10.7)	0.3	0.009
**Results without significant difference**								
EGF	8.5 (7.3–10.7)	9.0 (7.6–10.0)	0.6	9.5 (8.3–10.2)	1.0	8.0 (7.1–9.2)	0.4	0.2
EOTAXIN/CCL11	10.2 (8.0–10.8)	9.4 (8.9–10.4)	1.0	9.5 (9.0–10.5)	0.9	9.3 (8.0–10.4)	1.0	0.8
GMCSF	13.5 (12.8–16.0)	13.0 (11.4–16.1)	0.7	12.0 (11.0–17.0)	0.4	14.0 (13.0–16.0)	0.8	0.4
IFN-α2	9.0 (7.3–11.0)	8.0 (7.7–10.0)	0.7	8.0 (7.9–8.0)	0.6	9.0 (7.2–11.0)	0.9	0.7
IFN-γ	12.2 (11.5–13.2)	11.9 (10.5–13.6)	0.5	13.4 (10.5–14.0)	0.5	11.0 (10.3–12.3)	0.09	0.08
IL-12p40	15.5 (12.7–17.6)	13.8 (11.6–18.0)	0.6	16.8 (12.2–21.5)	0.6	13.0 (11.5–14.0)	0.1	0.2
IL-12p70	8.6 (7.3–9.2)	9.0 (8.2–9.6)	0.3	9.5 (8.4–9.6)	0.2	8.4 (8.0–9.4)	0.6	0.2
IL-15	22.4 (18.2–28.1)	23.3 (19.6–25.9)	0.9	23.5 (20.0–28.5)	0.7	23.0 (18.5–25.0)	0.8	0.7
IL-17A	11.5 (10.2–12.3)	12.1 (10.2–12.6)	0.5	12.0 (10.1–13.0)	0.6	12.2 (10.2–12.5)	0.6	0.9
IL-1β	8.3 (7.3–9.3)	8.5 (8.1–9.5)	0.5	9.0 (8.0–9.5)	0.3	8.3 (8.1–8.6)	1.0	0.3
IL-2	13.9 (13.0–15.1)	13.3 (12.7–14.3)	0.3	13.0 (12.8–14.2)	0.3	13.9 (10.5–14.4)	0.4	0.8
IL-3	10.7 (9.5–11.0)	10.7 (10.3–11.5)	0.6	10.6 (10.4–11.5)	0.9	10.8 (10.1–11.5)	0.6	1.0
IL-5	8.9 (7.7–9.6)	9.5 (8.0–10.7)	0.4	10.5 (7.9–10.8)	0.1	8.5 (8.0–10.0)	1.0	0.3
IL-6	14.3 (12.7–17.3)	13.1 (10.9–15.5)	0.2	13.7 (11.0–15.3)	0.4	12.5 (10.5–16.0)	0.3	0.8
IL-8	1274.5 (842.8–2227.4)	1108.8 (509.4–2166.8)	0.6	1063.0 (531.0–1383.0)	0.4	1666.5 (444.5–4307.5)	1.0	0.5
MIP-1α/CCL3	23.8 (15.5–54.0)	16.5 (15.2–29.5)	0.3	16.0 (15.3–37.0)	0.6	17.0 (15.0–24.0)	0.3	0.7
MIP-1 β/CCL4	17.2 (14.0–24.3)	14.8 (12.9–18.5)	0.3	15.0 (12.5–18.0)	0.5	14.4 (13.0–20.0)	0.4	0.9
TNF-α	12.9 (10.7–17.3)	12.8 (8.8–14.6)	0.3	13.5 (8.0–15.0)	0.6	11.5 (9.0–14.5)	0.2	0.6
VEGF	12.8 (11.3–13.8)	13.3 (11.0–14.6)	0.7	13.5 (12.8–13.9)	0.4	11.5 (10.5–15.0)	0.8	0.7
IL-10	9.1 (7.9–9.9)	8.8 (8.4–10.4)	0.9	9.5 (8.6–13.6)	0.2	8.7 (7.0–8.8)	0.3	0.08
IL-13	11.7 (10.5–13.1)	11.7 (10.5–12.6)	1.0	11.9 (10.1–12.5)	0.9	11.6 (10.5–13.5)	0.9	0.8
IL-1RA	14.9 (13.9–18.0)	15.3 (13.8–17.4)	0.9	15.0 (13.0–19.0)	0.9	15.5 (14.1–16.0)	0.9	0.7
**CSF inflammatory parameters ratios (median [p25th-p75th]) with significant difference**								
IL-4/GCSF	0.6593 (0.5118–0.7775)	0.7846 (0.7281–0.9308)	0.01	0.8000 (0.7286–1.0000)	0.02	0.7636 (0.7267–0.8889)	0.09	0.6
IL-4/IL-1α	0.3468 (0.2348–0.4706)	0.5945 (0.4059–0.7702)	0.004	0.5756 (0.3333–0.7879)	0.05	0.6133 (0.4200–0.7643)	0.007	0.7
IL-4/IL-7	0.7637 (0.7014–0.8494)	0.9173 (0.7480–1.0708)	0.06	0.9709 (0.8870–1.2366)	0.02	0.7619 (0.7368–1.0444)	0.5	0.3
IL-4/IP10	0.008215 (0.004132–0.01604)	0.02426 (0.006650–0.05306)	0.02	0.02092 (0.004347–0.05000)	0.3	0.04092 (0.01749–0.06225)	0.009	0.4

^a^Comparison between cases with and without microcephaly and controls.

^b^Comparison between cases with microcephaly and controls.

^c^Comparison between cases without microcephaly and controls.

^d^Comparison between cases with and cases without microcephaly.

### Inflammatory imbalance in CSF of ZIKV-exposed neonates with microcephaly

**Figure 1:**
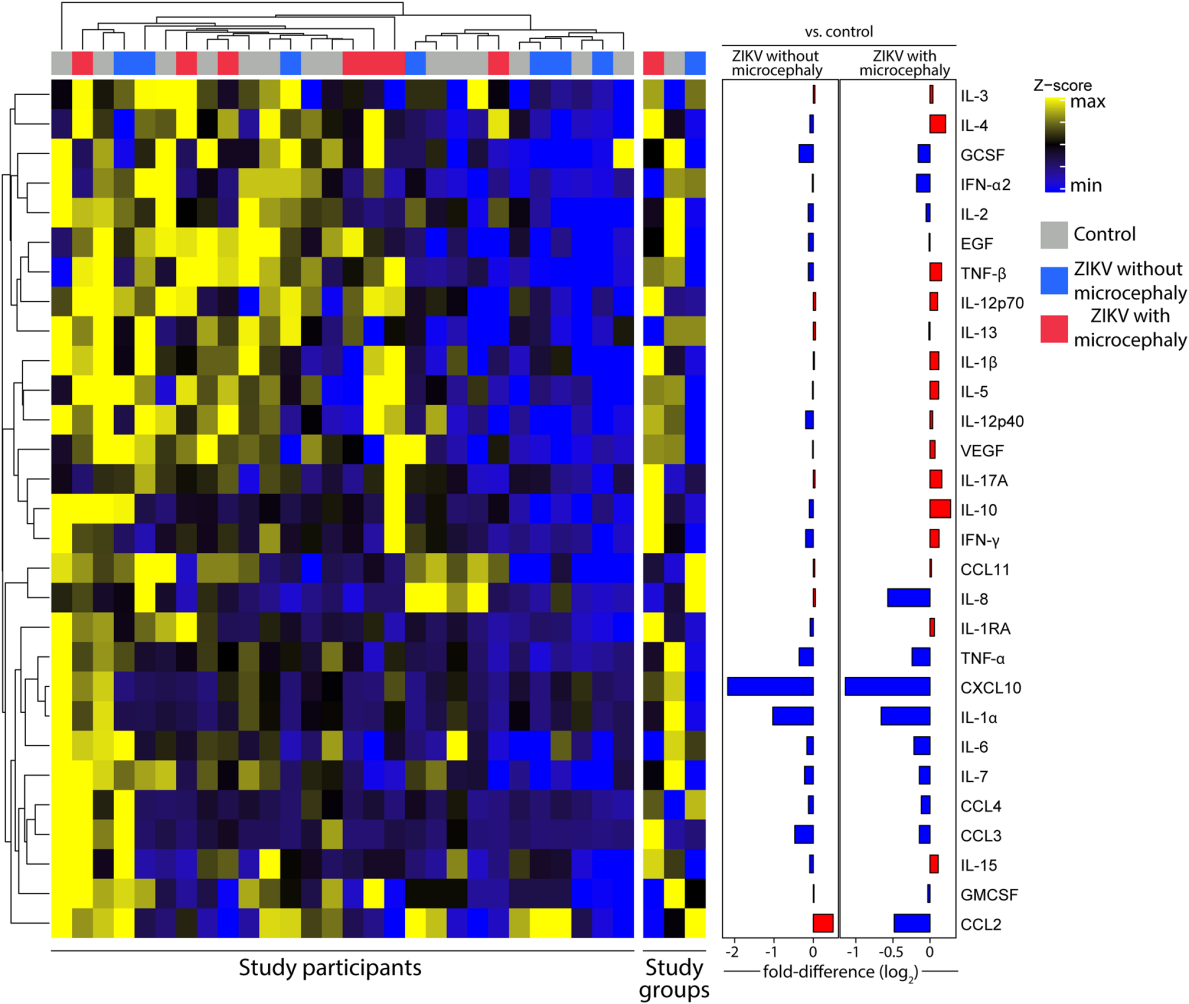
Patients exposed to Zika virus during fetal life and born with microcephaly express distinct inflammatory profile in cerebrospinal fluid: *Left panel*: Data were log-transformed and z-score normalized. A heatmap was built to describe the overall expression profile of the inflammatory markers measured in cerebrospinal fluid (CSF) in each study group labeled according to the experimental group indicated by distinct colors (gray control, blue Zika virus without microcephaly and red Zika virus with microcephaly). *Right panel*: Average fold-difference values in expression of inflammatory markers in CSF from children with zika virus exposure, zika virus exposure that developed microcephaly and control group are described (log-transformed values). Red bars infer markers which values tended to be higher in the disease groups whereas blue bars denote markers which concentrations were higher in the control group.

**Figure 2:**
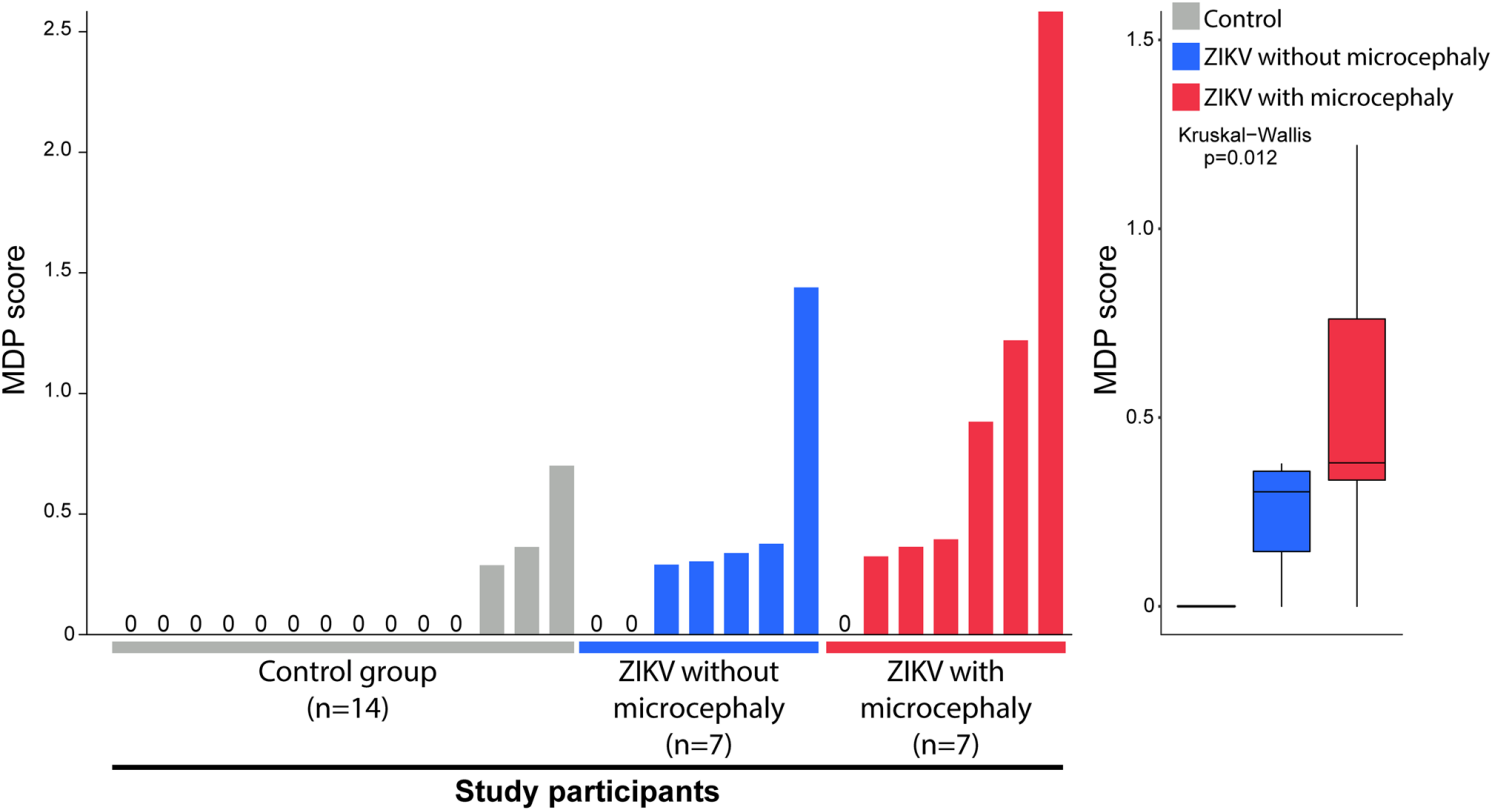
Children that developed microcephaly exhibit higher molecular degree of perturbation in CSF. *Left panel:* Histograms show the single sample molecular degree of perturbation (MDP) score values relative to each study group as indicated. MDP values were calculated as described in Methods and in Oliveira-de-Souza et al.^36^. *Right panel:* Box plots represent the distribution of the MDP among study groups. Values were compared among the control, ZIKV-exposed with or without microcephaly groups using the Kruskal–Wallis test with Dunn’s multiple comparisons. All the differences between the groups were statistically different (p < 0.05). In the box plots, lines represent median and interquartile range values.

To access the inflammatory changes in CSF related to CZI, we examined the expression of 29 inflammation mediators soluble proteins, stratified according to presence/absence of microcephaly in neonates exposed to ZIKV during pregnancy and in controls (Fig. 1). Unsupervised hierarchical cluster analysis demonstrated that ZIKV-exposed individuals with microcephaly exhibited a tendency of increased levels of pro-inflammatory mediators in CSF, as IL-1β, IL-12p70, IL-15, IL-17A, IFN-γ, and MIP-1α (CCL3) compared to controls. Conversely, ZIKV-exposed participants without microcephaly displayed a general trend to decrease concentrations of inflammation mediators as IL-2, IL-4, IL-12p40, IL-12p70, TNF-α, TNF-β, IFN-γ, and the growth factors GCSF and EGF (Fig. 1). Furthermore, we employed a fold-difference analysis between controls and ZIKV-exposed individuals with or without microcephaly. In the ZIKV-exposed group without microcephaly, we identified a predominance of decreases in most parameters related to controls, except for MCP1 (CCL2) levels, that were significantly increased. Notably, microcephalic neonates displayed an increase of IL-1RA, IL-1β, IL-3, IL-4, IL-5, IL-10, IL-12p70, IL-12p40, IL-15, IL-17A, TNF-β, IFN-γ, and VEGF compared to controls. Additionally, fold differences were in general higher in ZIKV-exposed microcephalic cases compared to controls, suggesting a higher degree of inflammatory disturbance in CSF. To test this hypothesis, we calculated the MDP (Fig. 2) adapted to protein measurements. Using this approach, neonates with ZIKV-associated microcephaly presented substantially high degree of inflammatory perturbance, reinforcing that microcephaly is linked to marked inflammatory imbalance in CSF (Fig. 2).

### ZIKV infection leads to reduction in correlations between CSF levels of inflammation mediators

**Figure 3:**
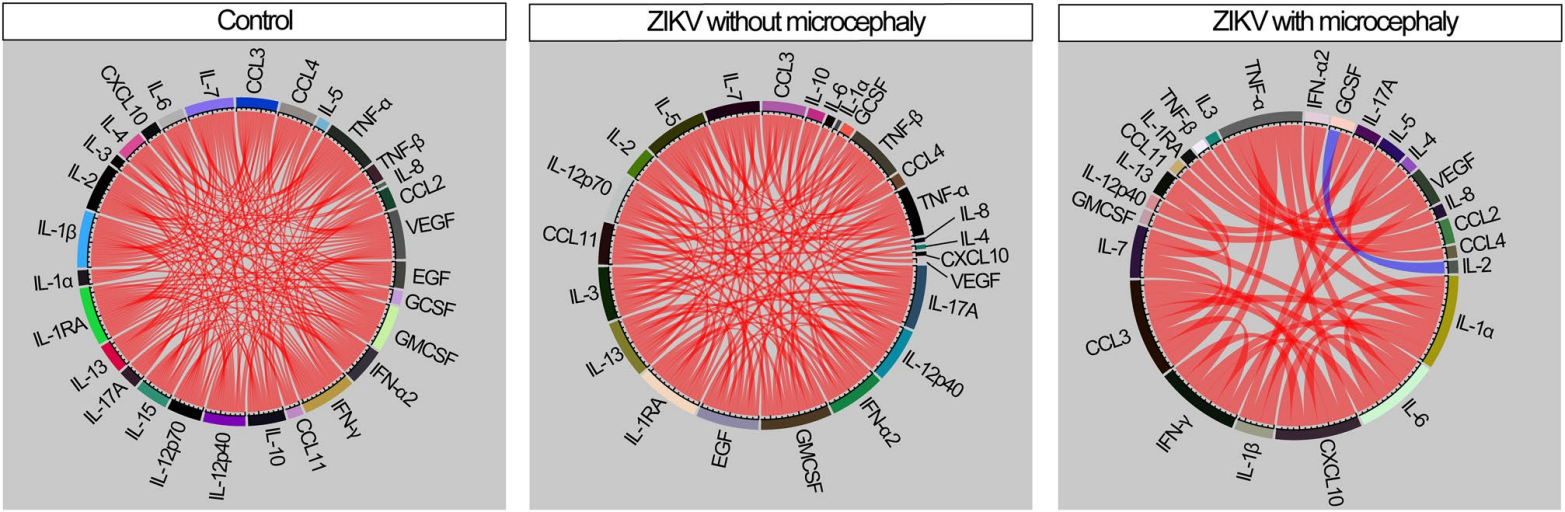
Presence of Zika virus infection leads to reduction in correlations between CSF levels of mediators of inflammation. Spearman correlation analysis was used to test association between cytokine values in CSF in control group (*left panel*) and in those infection patients who were only Zika infection (central panel) or Zika virus infection and microcephaly (*right panel*). Bars represent the Spearman rank (rho) values. Colored bars indicate statistically significant correlations (P < 0.05) after adjustment for multiple measurements. Red color infers positive correlation whereas blue color denotes negative correlations.

The inflammatory process is very dynamic, with synchronized changes in production and secretion of inflammation mediators influenced by host immunological status and pathogen load and virulence. To characterize this process, we have been using Spearman correlation networks that identify the dynamic changes in quality and degree of inflammation according to study groups^11–14^ (Fig. 3). By doing this in the present study, we found that the highest density of significant correlations was found in CSF of controls, whereas ZIKV-exposed microcephalic cases had the lowest number of interactions (Fig. 3). Regardless of the clinical groups, most of the significant correlations were positive, meaning that increased concentrations of a given marker were mostly followed by elevated levels of other inflammatory molecules. Importantly, a unique negative significant correlation was found between IL-2 and GCSF in the group of ZIKV-associated microcephalic cases (Fig. 3).

Noteworthily, in the control group, network analysis showed that most of the biomarkers measured in CSF presented a similar influence in density of correlation matrices, indicating that homeostasis may result from coordinated response. Despite the similarity in the participation of connections between all markers, the most highly connect marker was IL-1RA, followed by IL-1β, IFN-γ, GMCSF and VEGF, all displaying only positive correlations (Fig. 3). In ZIKV-exposed neonates without microcephaly, GMCSF is the top node, also with only positive interactions, followed by pro-inflammatory cytokines such as IL-1RA, IL-12p70, IL-17A, TNF-α and IFN-α (Fig. 3). Furthermore, in ZIKV-associated microcephalic neonates, we could observe a significant decrease in number of correlations and increase in quantity of parameters accumulating strong connections, such as IL-1α, IL-6, TNF-α, IFN-γ, MIP-1α (CCL3) and IP10 (CXCL10) (Fig. 3). Thus, these findings argue that ZIKV-induced microcephaly is associated with uncoupled CSF inflammatory response, characterized by decreases in correlations between concentrations of inflammatory biomarkers.

### The correlation profile of concentrations of CSF inflammatory markers can distinguish neonates with or without microcephaly and probable CZI from uninfected controls

**Figure 4:**
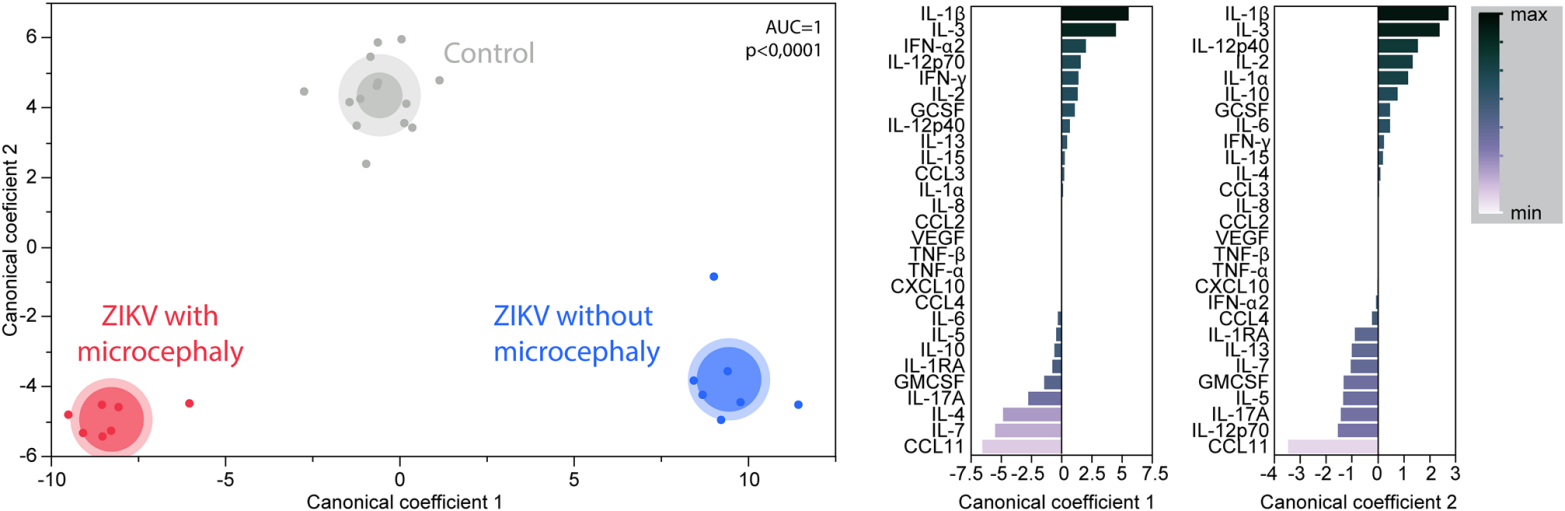
Discrimination of groups using combination of inflammatory biomarkers in cerebrospinal fluid: *Left panel:* In an exploratory approach, a sparse canonical correlation analysis (sCCA) was employed to test whether experimental groups could be distinguished based on the overall expression profile of all the markers measured. *Right panel:* Canonical coefficient scores were calculated and ranked to identify the biomarkers responsible for the difference between groups in the sCCA model.

Finally, we tested whether simultaneous measurements of CSF inflammation biomarkers could be used to distinguish the clinical groups. We employed a discriminant model using canonical correlation analysis, as we found distinct correlation profiles between the concentrations of biomarkers, and the model takes account the number, strength and parameters involved in correlations between biomarkers concentrations^12,15^. We found that the groups of ZIKV-exposed neonates, with or without microcephaly, could be completely distinguished from the control group (area under curve [AUC] = 1; p < 0.0001) (Fig. 4). Next, we plotted the canonical coefficient score of each biomarker included in the model to identify the top markers associated with the discrimination among groups. In the canonical coefficient analysis between controls and those exposed to ZIKV without microcephaly, we found that IL-1β, IL-3, IL-4, IL-7 and EOTAXIN (CCL11) were the top CSF markers in our model. When the groups of neonates with microcephaly and controls were compared, we identified the top discriminant scores for IL-1β, IL-3, EOTAXIN (CCL11) and IL-12p70. These findings suggest that ZIKV infection and occurrence of microcephaly leads to unique disturbances in systemic inflammation and immune activation profile that hallmark this condition.

## Discussion

Notably, CSF inflammatory parameters are modified in neonates exposed to ZIKV infection during pregnancy, according to presence or absence of congenital microcephaly. To the best of our knowledge, this is the first time in which such findings are reported from ZIKV-exposed neonates with and without microcephaly and controls.

The mechanism by which ZIKV invades CSF crossing the placental and blood–brain barriers has been elucidated^16^. Nevertheless, the immunopathologic pathways driven by CSF infection which leads to microcephaly remain poorly understood. We performed a detailed investigation of the inflammatory and immune activation profile in CSF of neonates with probable CZI.

Our exploratory analyses demonstrated the CSF inflammatory profile from neonates with ZIKV-associated microcephaly and delineated a biosignature able to distinguish ZIKV-exposed neonates from uninfected controls. In a Flavivirus infection model, investigations described a cytokine storm with immunological imbalance, with a pro-inflammatory response detected in acute phase^17,18^. Herein, neonates with ZIKV-associated microcephaly exhibited general increase in concentrations of pro-inflammatory mediators compared to controls. These neonates presented higher levels of IL-1β, IL-4, IL-10 and VEGF as previously shown in the Flavivirus model^17^, in addition to IFN-γ. The IFN pathway has been associated with protection of trophoblastic and non-trophoblastic cells from CZI^19^ and with viral replication in human placental macrophages^20^, with higher expression of type I IFN. Despite possible antiviral response mediated by IFN to ZIKV, other important IFN-related cytokines, as IP10 (CXCL10) levels had tendency to decrease in our analysis in both neonates with or without microcephaly, whereas concentrations of IFN-α2 displayed a tendency to decrease in CSF from microcephalic neonates and increase in those without ZIKV-associated microcephaly. Neonates with microcephaly possibly express other myeloid activation signals resulting in disturbances in the IFN pathway. Additional mechanistic studies are needed to test this hypothesis.

The association between microcephaly development and immune activation remains unclear, and the influence of inflammatory changes in such process is still unknown. The presence of ZIKV infection leads to alteration in gene regulation associated with immunological homeostasis, including cell cycle, differentiation and apoptosis in neuronal progenitor cells^21,22^. Reduction in cell growth which culminates in neurological malformations such as microcephaly was observed in an experimental model^22^. Besides that, the CNS may be directly affected due to an unbalanced local inflammatory response elicited by ZIKV infection which contributes to fetal brain damage^23^. Results from an immunogenetical study that looked to factors associated with the host phenotype against ZIKV infection suggested that variations at Toll-like receptor 3 and TNF-α genes associated with innate immune responses in pregnant mothers infected with ZIKV and their CZI babies may influence the risk of occurrence of microcephaly^24^. However, the specific interplay between immunologic factors and microcephaly development remains unclear. Our results revealed that the concentration values of inflammatory markers in CSF alongside the relationships among them are altered in ZIKV-associated microcephaly.

Using network analysis of Spearman correlation matrices, we demonstrated consistent changes in inflammatory dynamicity according to clinical presentation. ZIKV exposure during fetal life was associated with important differences in the network connectivity profile, involving both the quality and strength of correlations between biomarkers. Importantly, we found a decreased network density in both groups with probable CZI. Other analyses from our group suggest that changes in network presentation indicate alteration in systemic inflammation in other scenarios^11–14^. In this setting, increases in network connectivity usually infer augmented inflammation whereas decreases in the number of correlation or presence of negative associations may indicate uncoupling of the immune activation and leading to an imbalanced inflammatory response. In agreement with this idea, Vinhaes et al. recently reported that there is also an inflammatory imbalance in umbilical cord blood of neonates with CZI, and that the magnitude of the immune activation perturbation was inversely correlated with the cephalic perimeter^10^. Interestingly, in direct contrast with the results presenting here, the network analysis of concentrations of inflammatory mediators reported by Vinhaes et al. showed increased density (inferring the number of statistically significant correlations) in neonates with ZIKV-microcephaly^10^, whereas in the present study in CSF we found a reduction in connectivity of the inflammatory networks. This discrepancy between results from blood vs. CSF could be consequence of the differential activation of immune responses in distinct compartments, which may involve tissue-specific regulation of immune responses^25^. Those results, together with the findings of our present study, make us hypothesize that the hyperinflammatory milieu examined by MDP assessment in CSF may represent the immune activation at the CNS driving cytotoxicity related to immunopathology^26,27^, further leading to microcephaly. Future studies are needed to direct test our hypothesis in experimental pre-clinical models in which immune modulators could be evaluated as an approach to prevent extensive damage of the CNS and microcephaly. Neonates with probable CZI with microcephaly had strong elevation of the IgM and IgA classes in CSF^28^. Along with the significant increase in IL-4 levels in CSF of microcephalic neonates described here (Table 1), it is possible that an exacerbated activity of antibody-producing cells driven by this cytokine may take place in ZIKV-associated microcephaly. Additional investigations merging microbiologic and immunologic data are warranted to address this matter.

An important contribution of our study was the assessment of degree of immunological perturbation in CSF of neonates exposed to ZIKV during fetal life. To our knowledge, no previous study has estimated the global inflammatory disturbance in CSF of neonates with microcephaly. CSF of neonates with ZIKV-associated microcephaly exhibited higher degree of inflammatory disturbance in response to infection. Inflammation is part of a response focused on reestablishment of homeostasis, but if it is exaggerated/dysregulated, this process can result in inadequate tissue remodeling and cellular damage which are associated with unfavorable clinical outcomes in the context of several infectious diseases, such as tuberculosis^29–31^ and HIV/AIDS^32^. The role of inflammation is clear in traumatic brain injury^33^, but it remains unclear in neurological infections.

Importantly, the canonical model based on discriminant analysis described here identified possible candidate markers likely responsible for the discrimination between uninfected neonates and CZI with or without microcephaly, in CSF. Hence, IL-1β, IL-3, IL-12p70 and EOTAXIN (CCL11) appeared in our canonical coefficient model as top markers in the discrimination between both ZIKV-exposed groups and uninfected controls, whereas IL-4 and IFN-α2 appeared to be important only in distinction between those without microcephaly *vs.* controls and IL-17A in microcephalic neonates vs. controls. Of note, some of the top markers identified in a similar discrimination analysis obtained from umbilical cord blood were similar to those described here in the comparisons between ZIKV-exposed participants without microcephaly and controls, such as IFN-α2 and IL-1β^10^, indicating that these markers may play relevant role in both peripheral blood and CNS. In addition, reproducing the idea reported herein, the previously published results from umbilical cord blood also indicated that the inflammatory profile induced by exposure to ZIKV is very distinct and can characterize ZIKV-microcephaly^10^. Our observations suggest that the immune activation profile detected in CZI was so dramatically different that could distinguish infection with or without microcephaly with high degree of accuracy, highlighting possible biomarkers directly associated in the response of ZIKV infection and with the pathogenesis of microcephaly. Altogether, these findings also reinforce the role of tissue-specific inflammatory activation in the context of ZIKV infection.

Our study has some limitations. We examined samples obtained from a single time-point, which preclude us from making conclusions about the dynamicity of inflammatory process before birth. The number of individuals investigated was small, but the groups were carefully matched to reduce potential confounding factors. In addition, we have employed multidimensional analyses that have been used for analysis of small datasets as well. We adjusted the results for multiple measurements to minimize the odds of spurious findings. Finally, enterovirus infection was not investigated either in cases or in controls. However, CSF was obtained from all included neonates before maternity unit discharge what makes enterovirus infection improbable.

In conclusion, our finds extend the current information about CZI and immune activation status of CSF in pediatric patients, with delineation of the inflammatory changes associated with microcephaly. The degree of inflammatory imbalance may be associated with microcephaly driven by cytotoxicity in CZI and it may aid additional investigations in experimental pre-clinical models testing immune modulators in preventing extensive damage of the CNS. Additionally, they can be applied to the prospective evaluation of neonates with other viral congenital infections^34^.

## Methods and patients

### Ethics approval

The study was approved by the Ethics Committee of the Federal University of Bahia under the number CAAE: 59198016.2.0000.5577. All research was performed in accordance with the relevant guidelines and regulations. Mothers were interviewed after informed consent had been signed.

### Study design

This is a case–control study with a retrospective and a prospective component. Neonates exposed (cases), with or without microcephaly, and not exposed (controls) to ZIKV during pregnancy, who underwent lumbar puncture (LP) in the CSF Laboratory, in Salvador, Brazil, were evaluated. All LPs were performed at the discretion of the neonatologist. CSF was obtained from a LP in cases in the beginning of the ZIKV epidemic in Brazil (December/2015-March/2016) due to congenital infection clinical characteristics. The clinical epidemiological criteria to diagnose probable CZI included (1) LP performed during the ZIKV infection epidemic plus (2) ZIKV symptoms during pregnancy reported by mothers resident in the ZIKV epidemic affected area plus (3) negative laboratory tests to investigate other congenital infections (Syphilis, Toxoplasmosis, Rubella, Cytomegalovirus, Herpes, HIV, HTLV, Hepatitis B and C [STORCH-HIV-HTLV-HB-HC]). CSF was obtained from a LP in controls between November/2017 and September/2018. CSF was obtained from all included neonates (cases and controls) before maternity unit discharge. All patients were identified in the CSF Laboratory logbook. After reviewing the CSF charts, the medical charts were reviewed, and the respective mothers were interviewed, after informed consent had been signed, to collect prenatal and neonatal data. Information about serological tests (STORCH-HIV-HTLV-HB-HC) from mother and child was searched for, as well as information about neonates’ age, gender, weight, gestational age, 5-min Apgar, head circumference and neuroimaging. After confirmation of patients’ eligibility, the residual CSF kept frozen at − 20 °C was transported to the Gonçalo Moniz Institute (FIOCRUZ), to measure inflammation mediators.

### Cases

Sixteen neonates were identified. All mothers reported clinical symptoms from the ZIKV infection during pregnancy (rash, fever, myalgia, arthralgia), 13 (81.3%) during the first (≤ 13 weeks) and 3 (18.7%) during the second trimester (14–25 weeks). Congenital microcephaly was defined as head circumference lower than 31.9 cm (boys) and 31.5 cm (girls) for full-term (≥ 37 weeks of gestation) neonates and > 2 standard deviations below average for premature ones (gestational age < 37 weeks)^35,36^.

### Controls

Inclusion criteria comprised: age ≤ 4 days, CSF White Blood Cell (WBC) count ≤ 8/mm^3^, CSF protein ≤ 132 mg/dl, CSF Red Blood Cell (RBC) count ≤ 1000 cells/mm^3^, absence of CNS disease, any congenital infection and microcephaly. All controls had negative laboratory tests to investigate congenital infections (STORCH-HIV-HTLV-HB-HC) as well as negative blood, urine, and CSF cultures.

### Inflammatory profile in CSF

We evaluated 29 markers to examine the inflammatory and immune activation profiles. The cytokines interleukin (IL)-1α, IL-1β, IL-1RA, IL-2, IL-3, IL-4, IL-5, IL-6, IL-7, IL-8, IL10, IL-12p40, IL-12p70, IL-13, IL-15, IL-17A, interferon (IFN)- α2, IFN-γ, tissue necrosis factor (TNF)-α, TNF-β, granulocyte colony-stimulating factor (GCSF), granulocyte macrophage colony-stimulating factor (GMCSF), epidermal growth factor (EGF), vascular endothelial growth factor (VEGF), monocyte chemoattractant protein-1 [MCP-1 (CCL2)], interferon gamma induced protein/chemokine (C-X-C motif) ligand 10 [IP-10 (CXCL10)], macrophage inflammatory protein-1 alpha [MIP-1α (CCL3)], MIP-1 beta [MIP-1β (CCL4)] and EOTAXIN (CCL11) were measured using a commercial kit from Luminex (Millipore, Boston, MA) according to the manufacturer’s instructions.

### Data analysis

Data was entered in the Statistical Package for Social Sciences (version 9.0) software and checked twice to ensure correctness. The median values with interquartile ranges were used as measures of central tendency and dispersion. Comparison between two groups was performed by using Mann Whitney *U* test. The Kruskal–Wallis test with the Dunn’s multiple comparisons ad hoc test were used to compare continuous variables and the Pearson’s chi-square test was used to compare variables displayed as frequency/percentage. The Spearman rank test was used to identify correlations between different cytokines. Correlations with coefficient (rho) > 0.5 and P < 0.05 were included in the network visualization.

A hierarchical cluster analysis (Ward’s method), with 100X bootstrap of log10 transformed and Z-score (row) normalized data was employed to depict the overall expression profile of indicated biomarkers. In such analysis, dendrograms represent the Euclidean distance. All comparisons were two-tailed and pre-specified. Differences with P-values below 0.05 after Holm-Bonferroni’s adjustment for multiple comparisons were considered statistically significant.

The molecular degree of perturbation (MDP) in CSF measurements was calculated to infer the level of inflammatory imbalance associated with probable CZI. This method has been detailed previously^11,12,37^. MDP is a novel statistical approach proposed by us to evaluate the degree of inflammatory disturbance in patients with a variety of infectious diseases^11,12^. Healthy controls were defined as the “reference” group, and the average level and standard deviation of this reference group were calculated for the CSF concentrations of each inflammatory marker. The MDP score of an individual marker in a given sample “s” was defined by taking the difference in concentration level in sample “s” from the average of the marker in reference group divided by the corresponding standard deviation. The MDP score represents the number of standard deviations from the reference. Individuals who had MDP values > 2 standard deviations from mean value of controls were considered molecularly perturbed. A detailed description of each step used to calculate MDP values is shown in Fig. S1. P-values < 0.05 were considered statistically significant.

The statistical analyses were performed using GraphPad Prism 8.0 (GraphPad Software, La Jolla, CA), STATA 11 (StataCorp, College Station, TX), JMP 14.0 (SAS, Cary, NC) and Gephi (version 0.9.2) programs 0.82 with circular layout plug-in.

## Supplementary Information

Supplementary Information 1.

Supplementary Information 2.

## Data Availability

The dataset generated and analyzed during the current study is available from the corresponding author on reasonable request (CSF Dataset.xls).
