# Supplementary material for: Zika-exposed microcephalic neonates exhibit higher degree of inflammatory imbalance in cerebrospinal fluid
Source: Sci Rep. 2021 Apr 19;11:8474. doi: 10.1038/s41598-021-87895-4 (PMC8055905; doi:10.1038/s41598-021-87895-4)
Supplement: Supplementary file 1 — Supplementary Information 1. [file 41598_2021_87895_MOESM1_ESM.docx]

**Zika-exposed microcephalic neonates exhibit higher degree of inflammatory imbalance in cerebrospinal fluid**

Gustavo C. Nascimento-Carvalho^1,*^, Eduardo C. Nascimento-Carvalho^1^, Clara L. Ramos^1^, Ana-Luisa Vilas-Boas^1^, Otávio A. Moreno-Carvalho^2^, Caian L. Vinhaes^1,3,4^, Beatriz Barreto-Duarte^3,4,5^, Artur T.L. Queiroz^3,4^, Bruno B. Andrade^1,3,4,5,6,7,8,†^, and Cristiana C. Nascimento-Carvalho^9,†^

^1^Bahiana Foundation for Science Development, Bahiana School of Medicine, Salvador, Bahia, 40290-000, Brazil

^2^Cerebrospinal Fluid Laboratory, José Silveira Foundation, Salvador, Bahia, 40170-100, Brazil

^3^Gonçalo Moniz Institute, Oswaldo Cruz Foundation, Salvador, Bahia, 40296-710, Brazil

^4^Multinational Organization Network Sponsoring Translational and Epidemiological Research (MONSTER) Initiative, Salvador, Bahia, 40296-710, Brazil

^5^University Salvador (UNIFACS), Laureate Universities, Salvador, Bahia, 41820-021, Brazil

^6^School of Medicine, Faculdade de Tecnologia e Ciências (Uni-FTC), Salvador, Bahia, 41741-590, Brazil

^7^Wellcome Centre for Infectious Disease Research in Africa, Institute of Infectious Disease and Molecular Medicine, University of Cape Town, Cape Town, 7700, South Africa

^8^Division of Infectious Diseases, Department of Medicine, Vanderbilt University School of Medicine, Nashville, TN 37232, USA

^9^Federal University of Bahia School of Medicine, Department of Pediatrics, Salvador, Bahia, 40210-630, Brazil

*email: Gustavo C. Nascimento-Carvalho: [gcn.carvalho@hotmail.com](mailto:gcn.carvalho@hotmail.com)

^†^These authors contributed equally to this work.

**Supplementary Table S1** **Characteristics of 14 neonates (7 with and 7 without microcephaly) exposed to Zika virus during fetal life and 14 controls**

| Characteristic | All cases  (n=14) | Cases with microcephaly  (n=7) | Cases without microcephaly (n=7) | Controls  (n=14) |
| --- | --- | --- | --- | --- |
| Median (IQR) age (days) | 2 (1.0-3.0)  Oldest 4 | 1 (1-3) | 2.5 (1.3-3.0) | 3.0 (1.0-4.0)  Oldest 4 |
| Girls | 9 (64.3%) | 5 (71.4%) | 4 (57.1%) | 10 (71.4%) |
| Prenatal care | 14 (100.0%) | 7 (100.0%) | 7 (100.0%) | 14 (100.0%) |
| Premature | 3 (21.4%)  32w, 33w, 35w^a^ | 2 (28.6%) | 1 (14.3%) | 4 (28.6%)  27w, 29w, 32w, 34w^a^ |
| Microcephaly | 7 (50.0%) | 7 (100.0%) | 0 | 0 |
| Minimal 5-minute Apgar | 8 | 8 | 8 | 7 |
| Head neuroimaging^b^ | 13 (92.9%) | 6 (85.7%) | 7 (100.0%) | 7 (50.0%) |
| Abnormal findings | 9 (64.3%) | 6/6 (100.0%) | 3/7 (42.9%) | 0 |

^a^Gestational age in weeks.

^b^Head neuroimaging was performed in the controls with seizure (n=1) and sepsis (n=6) because maternity units protocol includes head ultrasound in the standard investigation of neonates with seizure and sepsis.

**Supplementary Figure S1**


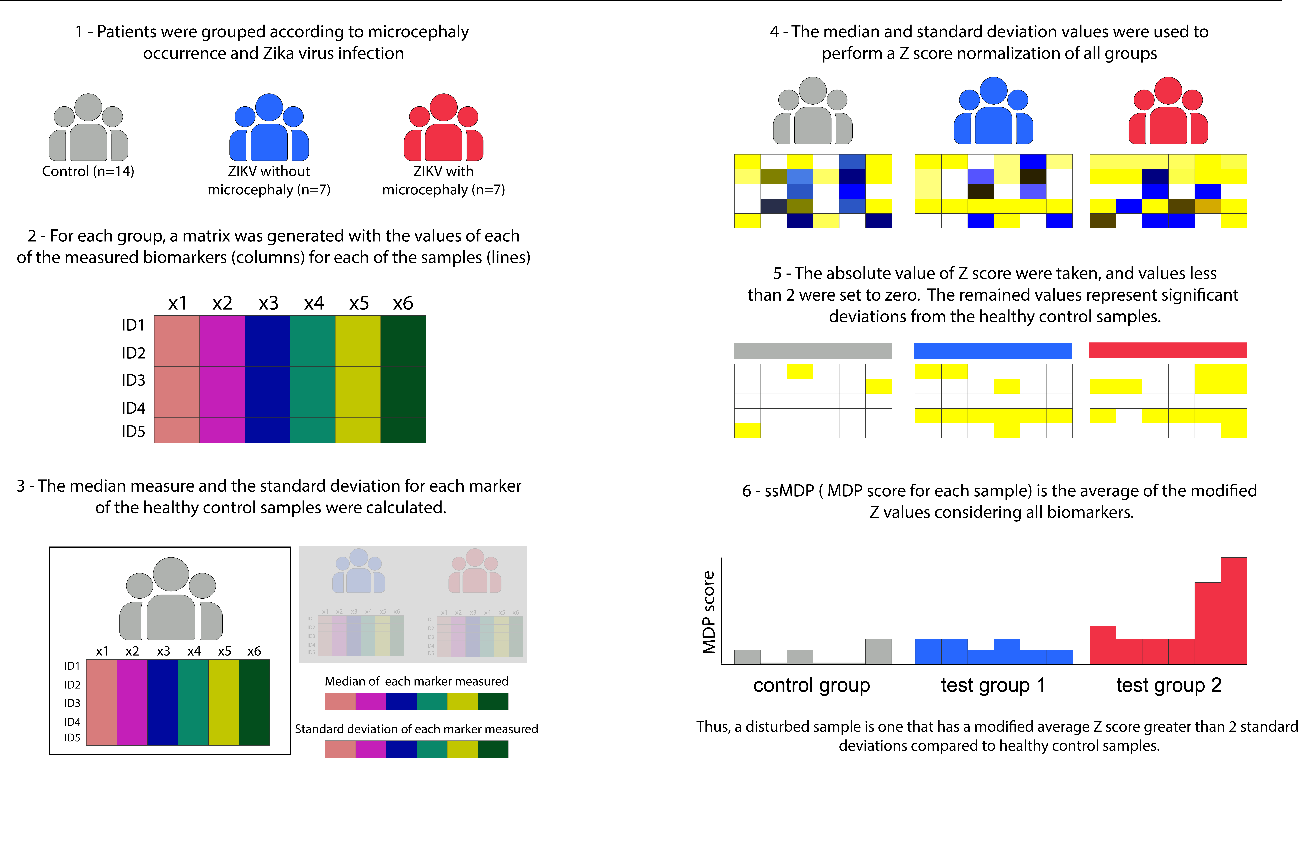


**Figure S1**. Step by step of adapted Molecular Degree of Perturbation, using biomarker measures, instead of gene expression, as described in Methods. (i) Patients were grouped according to microcephaly occurrence and ZIKV infection, (ii) and for each group was generated a matrix with biomarkers measures. (iii) The median and standard deviation (SD) values of each biomarker of the control group were calculated firstly. (iv) Then, the Z-score was calculated for all groups using these parameters, and (v) modified using a cut-off point established (<2 SD). (vi) Thus, an average disturbance calculation was performed for each sample resulting in the MDP score. This figure is adapted https://mdp.sysbio.tools/about.
